# Compared to self-immersion, mindful attention reduces salivation and automatic food bias

**DOI:** 10.1038/s41598-017-13662-z

**Published:** 2017-10-23

**Authors:** Constanza Baquedano, Rodrigo Vergara, Vladimir Lopez, Catalina Fabar, Diego Cosmelli, Antoine Lutz

**Affiliations:** 1 Lyon Neuroscience Research Center INSERM U1028, CNRS UMR5292, Lyon 1 University, Lyon, France; 2 School of Psychology, Pontificia Universidad Católica de Chile, Santiago, Chile; 3 Interdisciplinary Center for Neuroscience, Pontificia Universidad Católica de Chile, Santiago, Chile; 4 Biomedical Neuroscience Institute, Faculty of Medicine, Universidad de Chile, Santiago, Chile

## Abstract

Immersing ourselves in food images can sometimes make it feel subjectively real, as if the actual food were right in front of us. Excessive self-immersion into mental content, however, is a hallmark of psychological distress, and of several psychiatric conditions. Being aware that imagined events are not necessarily an accurate depiction of reality is a key feature of psychotherapeutic approaches akin to mindfulness-based interventions. Yet, it is still largely unknown to what extent one’s engagement with mental content, considering it as real, biases one’s automatic tendencies toward the world. In this study, we measured the change in subjective realism induced by a self-immersion and a mindful attention instruction, using self-reports and saliva volumes. Then, we measured behaviorally the impact of subjective realism changes on automatic approach bias toward attractive food (FAB) using an approach–avoidance task. We found a reduction in saliva volume, followed by a reduction in FAB in the mindful condition compared to the immersed condition. During the immersed condition only, saliva volumes, state and trait measures of subjective realism, and food craving traits were positively correlated with FAB values, whereas meditation experience was negatively correlated to it. We conclude that mindful attention instructions can de-automatize food bias.

## Introduction

Thoughts can sometimes seem “subjectively real”, as if the imagined event was happening in the moment. This process of being self-immersed in the contents of one’s mind has been called cognitive fusion^1^, reification^2^, absorption^3^, experiential fusion^4^, or subjective realism^5^. In contrast, being aware that thoughts or perceptions are mere representations, and not necessarily an accurate depiction of reality, is a process that has been labeled as phenomenological reduction^6^, decentering^7^, cognitive diffusion^1^, mindful attention^8,9^, or dereification^2^. Subjective realism is common in daily life. For instance, merely reading about or viewing attractive food items is sufficient to trigger activation in the gustatory and reward areas in the brain^10^ or to increase salivation^11^. Blood levels of ghrelin, a hormone responsible for physiological hunger, are also modulated by one’s mere belief in the caloric values of food items^12^. By contrast, becoming aware that one has self-immersed into attractive food stimuli, and that this experience is a mere transient mental event, reduces reward simulations and appetitive behavior^9^. In this study, we aim to extend this research by investigating how much one’s engagement with thoughts and perceptions, considering them as being real or not, biases one’s automatic approaching/avoidance tendencies toward them.

**Figure 1:**
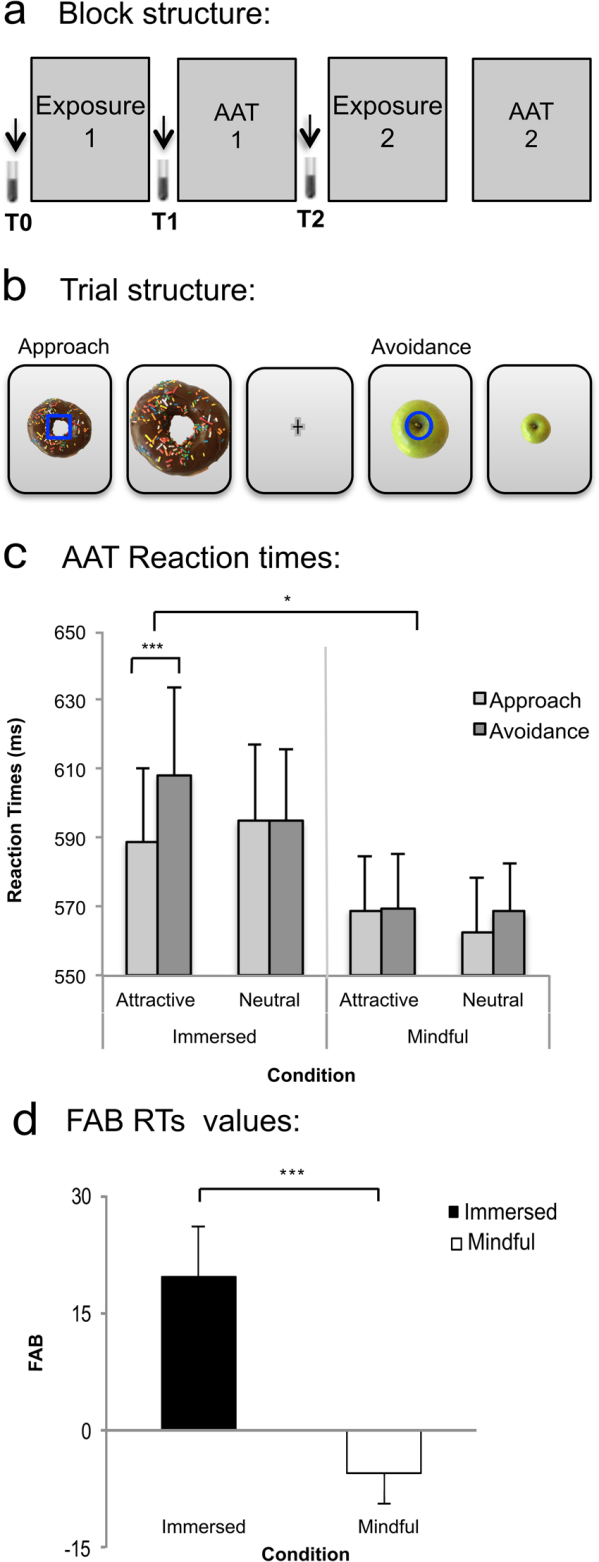
Experimental design and validation of the behavioral marker of automatic food bias: (**a**) Block structure: Participants observed food pictures following either a Mindful attention or an Immersion instruction (EXPOSURE 1) before performing an approach-avoidance task (AAT). We collected saliva samples before starting the experiment (T0), after the exposure phase (T1), and after the AAT (T2). After a 45-minute break, participants repeated this paradigm while following the remaining instruction. Finally, we classified the food images as neutral or attractive based on the participants’ ratings collected after the experiment. (**b**) Trial structure during AAT: To induce approach or avoidance tendencies toward food images, we overlaid one of two possible cues (a blue circle or square) on the food images, requesting the participants to move a joystick as quickly as possible, either toward them or away from them. Moving the joystick toward or away from them prompted the images to grow or shrink respectively. (**c**) Behavioral AAT Validation: Replicating Papies *et al*. (2012), we found a longer reaction time (in ms) in response to attractive food images compared to neutral food when an avoidance response was required compared to approach responses. We observed this during the Immersed condition, but not the Mindful condition. (**d**) FAB RTs were greater during Immersed condition than the Mindful condition. Error bars denote Standard Error. *p < *0*.05, ***p < *0*.001.

To address this question, we used a food engagement paradigm developed by Papies, Barsalou and Custers (2012)^8^, and adapted it to make it suitable for physiological measures (such as EEG, EKG, and salivation). In the aforementioned study, the authors applied an approach-avoidance task (AAT), in a between-subject design, to show that a mindful attention instruction decreases automatic impulses towards attractive food when compared to a control or immersion instruction. These two instructions manipulated the degree of engagement with mental events as being real or not. Neutral and attractive food images were displayed on a computer screen accompanied by a cue that indicated either to approach or avoid the presented food picture. The AAT paradigm implicitly assesses automatic approach-avoidance behavior^13^. The reaction times (RT) difference between incompatible conditions (i.e. avoiding attractive food) and compatible conditions (i.e. approaching attractive food) is referred to as the stimulus response compatibility (SRC) effect^14^. The SRC, which measures the specific AAT effect of food attractiveness, is labeled as the “Food Attractivity Bias” (FAB) (see Methods section for more details). Here we used a within-subject version of the task (see Fig. 1), where we presented the same set of neutral and attractive food images alongside both a mindful attention and an immersion instruction, presented in a randomized order across participants. With this design, we were able to measure how instruction-related changes in subjective realism influenced FAB, independently of the intrinsic saliency or reward of individual food cues.

In addition, to obtain a physiological indicator of immersion and dereification towards food, we measured salivary volume and salivary alpha-amylase (see Fig. 1). It is well known that exposure to food cues generates cephalic phase responses (CPRs), an autonomic anticipation reflex that prepares the organism to receive food. Among other indicators, this reflex is characterized by an increase in salivation^11^. To our knowledge, the relationship between amounts of salivation, and how strongly subjective food cues are perceived as real, has not been assessed before. Following this rational, we evaluated alpha amylase activity, which is one of the first enzymes acting on the digestive process, and is highly secreted in the presence of food^15^. Although this enzyme is mostly studied as a marker for sympathetic system activity, in this study we measured it to assess psychophysiological engagement toward food cues.

Through this study, we aim to refine our understanding of the cognitive processes underlying these two instructions, and their impact on food-related approach tendencies. Building on psychotherapeutic models of cognitive fusion^1^ and decentering^7^, and on phenomenological models of mindful attention^2,9^, we developed qualitative and phenomenological self-report measures along four sub-processes: craving, stickiness, meta-awareness, and dereification. It is necessary to deepen our understanding about these various constructs due to the lack of agreement regarding the overlaps and differences between them^7^. We validated these dimensions during the piloting of our paradigm (see^16^ and Methods and supplementary material for details). In the present framework, the immersion instruction should induce a state of high craving, high stickiness, low meta-awareness, and low dereification. The state of mindful attention should induce a state of low craving, low stickiness, high meta-awareness, and high dereification. Here we also assume that the particular contributions of these four sub-processes are modulated by the way these instructions are implemented, as well as by individual trait differences between these dimensions. To assess these processes as traits, we added the scale of cognitive fusion^17^, defined as a scale of low meta-awareness and high self-immersion, a scale of trait dereification, a scale of food craving, and the body-mass index of participants. We also recruited novices and experienced meditators among the participants, as the practice of mindfulness meditation is thought to develop the capacity of mindful attention.

We hypothesized that decreasing subjective realism towards attractive food pictures during a brief mindful attention instruction, compared to an immersion instruction (Fig. 1), would reduce the automatic tendencies toward attractive food compared to neutral food, as measured by reduced FAB. Secondly, we hypothesized that the instruction-related effects on FAB would be mediated by reduced salivation and alpha-amylase activities in response to food image exposure in mindful attention compared to immersion instructions. Finally, we hypothesized that the instruction-related effects on the FAB and saliva volume would be associated with the individual differences regarding the sub-processes of craving, stickiness, meta-awareness, and dereification, as measured by state and trait self-reports and by meditation experience.

## Methods

### Participants and recruitment

25 non-meditators (without prior experience in meditation practice) and 25 meditators (from 6 months to 6 years of experience in meditation, with a total of 1415 ± 1227 hours of practice), were recruited via flyers posted in universities, the location of various groups (football, volleyball, political, literature, dance, meditation, taekwondo and capoeira), and in two Buddhist meditation centers (the Shambhala center in the Vajrayana Tibetan tradition, and the Goenka center in the Theravada-Vipasssana tradition) in Santiago, Chile. The flyers did not explicitly mention that we were recruiting meditators to reduce self-selection group bias. We excluded participants with a self-reported history of psychological disorders (depression, anxiety, eating disorders), high body mass index (BMI, over 30 points) or any cardiac condition that could interfere with the aims of this study or put participants’ health at risk. The final sample consisted of 50 healthy adults: 25 (13 females) non-meditators of 28.7 ± 7.0 years of age, 3 of them vegetarians, and 25 (20 females) meditators of 28.6 ± 5.6 years of age, 9 of which were vegetarians (see supplementary materials for the psychometric descriptions of the two groups). All the procedures in this study were approved by the institutional Ethics Committee of the School of Psychology at the Pontifical Catholic University of Chile, in accordance the with guidance and regulation from the National Committee of Science and Technology of Chile (CONICYT). All participants gave informed consent prior to participation in the study.

### Task

We adapted a protocol of exposure to neutral and attractive food pictures, with either a mindful attention or immersed attitude instruction followed by an approach–avoidance task (AAT) as described in Papies *et al*. (2012) (see Fig. 1).

#### Exposure phase and instructions

Participants were asked to look at food pictures for 5 seconds while following two instructions given in randomized order across participants. The instructions are presented in the supplementary material. Briefly, during the mindful attention condition, participants were asked to “(…) be aware of thoughts, sensations and reactions while watching each image, and to experience these mental events just as mere constructions of the mind, that appear and disappear (…)”. During the immersed condition, participants were asked to “(…) try to connect intensely with the sensations generated by each image and get immersed into each image (…)”.

### Stimuli

#### Food image stimuli validation

To validate the food stimuli, an additional group of 100 Chileans ranked, via an online questionnaire, 300 items from the bank *Food-pics*, an image database for experimental research on eating and appetite^18^, according to their attractiveness on a 5-point Likert scale (from “very attractive” to “not attractive”). From the results of this questionnaire, we selected 60 images with the highest scores in the “very attractive” and “attractive” categories, and 60 images with the highest scores in “neither attractive nor unattractive” and “not attractive” categories to be displayed in the experiment.

#### Approach-avoidance task (AAT)

In this task, neutral and attractive food images were displayed with either a blue circle or a square centrally overlaid above the food item. Participants were asked to respond as quickly as possible, by moving a joystick toward them, or by moving it away from them, according to the cue provided, while RTs were collected. These movements correspond to the approaching- and avoiding the image conditions, respectively. Following the joystick movement, the pictures grew or shrank, mimicking approach or avoidance. Participants were allowed a maximum of 2 seconds to respond before the next image was displayed (Fig. 1 a,b).

#### Personal image pool construction

After having performed the exposure phase and AAT in the laboratory with the selected 120 food images, participants were asked to rate each one of these images on a Likert scale as described above. Personalized pools of attractive and neutral food images were computed from this information for each participant, to be used in the RT analysis.

### General Design and Procedure

#### General procedure

All experimental sessions started between 15:00 and 16:00 hrs. As a way to control for variations attributable to circadian rhythm on food intake and alpha amylase secretion^15^, participants were asked to a have a regular lunch at least one hour before coming in to the laboratory.

Upon arrival to the laboratory, participants were asked to fill out a “pre-state questionnaire” (see below and supplementary material for details). Participants then completed the exposure phase, where they were shown 60 food images, under either immersion or mindful attention instructions as previously described, followed by two blocks of the AAT. At the end of the experiment, participants were interviewed regarding their experience during the task using open and semi-structured questions. After the interview, participants filled out a “post-state questionnaire”. This sequence was performed twice, once for every instruction. Upon finishing the sequence under the first condition, participants had a break where a snack and herbal tea were offered in order to restore their initial state. After the break and snack, participants had 45 minutes of free time as a “psychological washout”, to allow physiological levels to approximate a comparable baseline. Then, participants filled out a new set of “pre-state questionnaires” and were invited to perform the same experimental sequence under the other instruction (the order of the instructions was counter-balanced across participants). Trait questionnaires, including food preference questionnaires, were sent by mail to be filled out at home just after the experimental session.

### Saliva Sampling and Alpha-Amylase Activity Measurement

Duplicated saliva samples were collected three times during the experimental sequence for each condition: before starting the experiment (“T0”, baseline sample), after the exposure phase (“T1” sample), and after the following AAT block (“T2” sample) (see Fig. 1a). Saliva was collected through passive drooling^19^ for one minute in a 5 ml cryotube, and immediately stored at −20 °C. Volumes were measured with a micropipette (Eppendorf, p1000) and alpha-amylase activity was measured by enzymatic assay with the “Salivary Alpha-amylase Kinetic Enzyme Assay Kit” (Salimetrics, LLC).

### Self-Report Measures

#### Trait questionnaires

In order to establish relations between trait characteristics and the instruction condition effects in the AAT, a battery of self-report questionnaires was applied after finishing the experimental lab setting. This battery was composed of the following questionnaires: Food-Craving Trait questionnaire^20^, Dereification-as-a-Trait questionnaire (constructed by us), Five-Facet Mindfulness Questionnaire^21–23^, and a Cognitive Fusion Questionnaire^17^ (Results of these questionnaires and comparison between groups can be found in supplementary results, Supp. Fig. 1a).

#### State questionnaire

Before starting each session, participants filled out a pre-state questionnaire, consisting of three Likert scale questions, to control for the role of mood and hunger in our experimental manipulation. In order to measure the effect of the instruction on the subjective experience, after participants performed the experiments in each instructed condition, they also filled out the post-state questionnaire. With this tool, we assessed four dimensions that we considered relevant for characterizing the first-person features of subjective realism in this paradigm. With the first two dimensions of “Dereification” and “Meta-awareness”, we aimed to directly assess self-perceived experience of subjective realism toward food. With the last two dimensions of “Craving” and “Stickiness”, we assessed the impulsive, and motivational consequences of subjectively perceived food images as real. Finally, an overall score reflecting how subjectively real items appeared was calculated based on the average scores for each sub-scale (more details and further examples along with this questionnaire validation are presented in the supplementary material).

### Statistical analysis

#### General design: independent variables

We considered 4 independent variables with two levels each: 1) Group: non-meditators versus experienced meditators; 2) Instruction type: mindful attention versus immersed; 3) Food type: appetizing versus neutral food items; 4) Response type: approach versus avoidance conditions. During the study, we measured the following dependent variables: 1) reaction times (RTs); 2) saliva volume; 3) salivary alpha-amylase activity; and 4) trait and state self-report questionnaire scores. All levels of statistical significance were set at *p* < 0.05.

#### Reaction times (RT) analysis

A 4 factor repeated-measures ANOVA was first conducted with Group as between-subject factor, and Instruction type, Food type and Response type as within-subject factors. To explore the relationship between RT and other dependent variables, we further reduced the number of within-subject factors related to RT by computing a “food attractiveness bias” (FAB) index as follows;

$$FAB=[Avoidanc{e}_{attractive}-Approac{h}_{attractive}]-Avoidanc{e}_{neutral}-Approac{h}_{neutral})]$$

where *avoidance* corresponds to RTs for avoidance responses, *approach* corresponds to RTs for approach responses, *attractive* being RTs for attractive food type and *neutral* being RTs for neutral food type. FAB is a representative value expressed in milliseconds, which gives account of the approach-avoidance tendencies toward each food type. The FAB value specifically expresses how much slower a person avoids than approaches attractive food images. One male subject was excluded from all analyses, including FAB, due to his FAB values being too high in the immersed condition, thus becoming an outlier (above three standard deviations) in relation to the group.

#### Saliva volume and Alpha-amylase analysis

Three-factor (two levels each) repeated-measures ANOVAs were conducted with Group as a between-subject factor and Instruction type and Time sample (T1/T2) as between-subject factors. The Time sample factor evaluated the fluctuations of salivary volume and alpha amylase activity across the paradigm (Fig. 1a).

#### Self-report analysis

We used paired t-tests to assess the differences between meditators and non-meditators on the trait questionnaires described above. For the state-sensitive questionnaires, we conducted for each sub-dimension (i.e. *dereification*, *meta-awareness*, *craving* and *stickiness*) two-factor (two levels each) repeated-measure ANOVAs with Group as between-subject factor, and Instruction condition as within-subject factor.

#### Integrative analysis

Once we determined the subjective realism modulation (i.e. behavioral, autonomic system and self-reports) in each one of the assessed dimensions, Pearson correlations and linear regression models were conducted to determine whether these measures were related to one another. Specifically, we evaluated whether self-report questionnaires (state and trait based) could predict RTs and autonomic measures, and whether RTs could predict autonomic measures.

## Results

### AAT Reaction Times (RTs)

We hypothesized that we would found behaviors consistent with the findings of Papies *et al*. (2012) regarding RTs during the AAT when the immersion and mindful attention instructions were given to each subject, instead of being compared between two different groups. We also hypothesized that the effect of the mindful attention instruction on the AAT, RT performances would be enhanced as a function of meditation experience. To test these hypotheses, we conducted a repeated-measures ANOVA, with Group as between-subject factor, and Instruction type, Food type, and Response type as within-subject factors.

We found a main Group effect, with meditators responding faster than non-meditators (Tables 1–2, Supp. Fig. 3). Also, we observed a main effect of Instruction type, where the RTs during the Mindful attention condition were faster than during the Immersion instruction (Tables 1–2, Fig. 1c). Finally, we observed a main effect of Response type, where approach RTs were overall faster than avoidance RTs (Tables 1–2, Fig. 1c). The first two effects were not predicted, and the third effect was similar to that described in Papies *et al*. (2012). We found a Food type x Response type interaction driven by slower RTs during avoidance compared to approach responses (t-test, *t*(49) = 2. 9 *p* < 0.01), which was specific for attractive compared to neutral food images (t-test, *t*(49) = 0.08 *p* = 0.4; Table 1, Fig. 1c). Consistent with our first hypothesis, we found an Instruction condition x Food type x Response type interaction. To further assess this three-way interaction, we examined the effects of Food type and Response type during Immersed and Mindful attention conditions in two separated ANOVAs (Tables 3–4).

**Table 1:** Approach –Avoidance Task Reaction times.

Group	Condition	Attractive food		Neutral food	
		Approach	Avoid	Approach	Avoid
Controls	**Immersed**	622 (147)	649 (180)	632 (155)	633 (149)
	**Mindful**	596 (112)	601 (110)	588 (108)	596 (101)
Meditators	**Immersed**	556 (95)	567 (97)	558 (106)	557 (100)
	**Mindful**	542 (94)	539 (85)	538 (89)	541 (87)

Reaction times and standard deviation (in ms) obtained during the AAT as function of Instruction, Group and Food type.

**Table 2:** Repeated-measures ANOVA, with Group as between-subject factor, and Instruction type, Food type, and Response type as within-subject factors.

Factor	DF	SSn	SSd	F	p	p < 0.05	ges
(Intercept)	1,48	1.35e + 02	4.27	1.52e + 03	5.01e − 38	*	9.63e − 01
Group	1,48	4.27e − 01	4.28	4.80e + 00	3.33e − 02	*	7.52e − 02
Condition	1,48	8.63e − 02	0.82	5.04e + 00	2.93e − 02	*	1.61e − 02
Response	1,48	4.71e − 03	0.05	4.51e + 00	3.88e − 02	*	8.95e − 04
Food: Response	1,48	1.24e − 03	0.01	4.07e + 00	4.90e − 02	*	2.37e − 04
Condicion:Food:Response	1,48	3.92e − 03	0.01	9.67e + 00	3.14e − 03	*	7.45e − 04
Group:Condition:Food:Respo	1,48	2.45e − 04	0.01	6.05e − 01	4.40e − 01	ns	4.67e − 05

**Table 3:** Repeated-measures ANOVA, in the immersed condition with Food type, and Response type as within-subject factors.

Factor	DF	SSn	SSd	F	p	p < 0.05	ges
(Intercept)	1,49	7.13e + 01	3.55	984.74	4.18e − 34	*	0.9512
Food	1,49	6.73e − 04	0.03	1.02	3.15e − 01	ns	0.0001
Response	1,49	5.03e − 03	0.047	5.19	2.70e − 02	*	0.0013
Food:Response	1,49	4.79e − 03	0.025	9.35	3.60e − 03	*	0.0013

**Table 4:** Repeated-measures ANOVA, in the Mindful Attention condition with Food type, and Response type as within-subject factors.

Factor	DF	SSn	SSd	F	p	p < 0.05	ges
(Intercept)	1,49	6.45e + 01	1.984	1592.35	4.99e − 39	ns	0.9692
Food	1,49	6.37e − 04	0.013	2.23	1.41e − 01	ns	0.0003
Response	1,49	6.84e − 04	0.038	0.87	3.56e − 01	ns	0.0003
Food:Response	1,49	3.74 e − 04	0.009	1.87	1.78e − 01	ns	0.0001

DFn: Degrees of Freedom in the numerator (a.k.a. DFeffect). DFd: Degrees of Freedom in the denominator (a.k.a. DFerror). SSn: Sum of Squares in the numerator (a.k.a. SSeffect). SSd: Sum of Squares in the denominator (a.k.a. SSerror). F: F-value. p: p-value (probability of the data given the null hypothesis). p < 0.05 Highlights p-values less than the traditional alpha level of 0.05. ges Generalized Eta-Squared measure of effect size.

In the Immersed condition ANOVA, we found a food positive bias, consistent with the observed interaction of Food type and Response type (Table 3). This interaction was driven by faster approach responses than avoidance responses toward attractive food images (t-test, *t*(49) = 3.6 *p* < 0.001), but not for neutral food images (t-test, *t*(49) = 0.04, *p* = 1.0). In contrast, there was no bias in the Mindful attention instruction, in agreement with the lack of Food type x Response type interaction during this condition. Finally, to explore the robustness of the 3-way interaction on RT, we ran both split-half reliability analysis with a random split, and permutation analysis procedure. These analyses confirmed our main finding.

We replicated this analysis on the Food Attractiveness Bias (FAB), and confirmed that the FAB values were greater in immersed than mindful conditions (M = 15, SD = 32.3, M = −5, SD = 28.4, respectively, t-test, *t*(48) = 3.1 *p* < 0.01) (Fig. 1d). This finding was not affected by the instruction order effect (see Supplementary Material, Supp. Fig. 4). We ran a split-half reliability test with our AAT scores (FAB) and obtained a Spearman-Brown coefficient of 0.505 when assessing the full sample. We detected two outliers in our split-half correlation, and removed them from the sample of the split-half reliability test, obtaining a Spearman-Brown coefficient of 0.78. Next, we re-ran our main ANOVA without these two participants, which corroborated that our main finding (i.e. the 3-way interaction on RT of Condition X Food X Response) was still present, (F (1,46) = 9, *p* < 0.05, η^2^_G_ = 0.0009). This indicates that the effect was still significant when removing these two outlier participants from the split-half reliability test, which is consistent with Papies *et al*’s (2012) findings.

To summarize, the mindful attention instruction condition, but not the immersion instruction condition, down-regulated the automatic approach-oriented impulses toward attractive food images (Fig. 1c and d), and this effect was reflected in the FAB index. Finally, contrary to our second hypothesis, we did not detect a four-way Group x Instruction condition x Food type x Response type interaction, suggesting that the effect of the Instruction condition on the Response type x Food type interaction was independent of meditation experience (Table 2).

### Salivary Volumes

**Figure 2:**
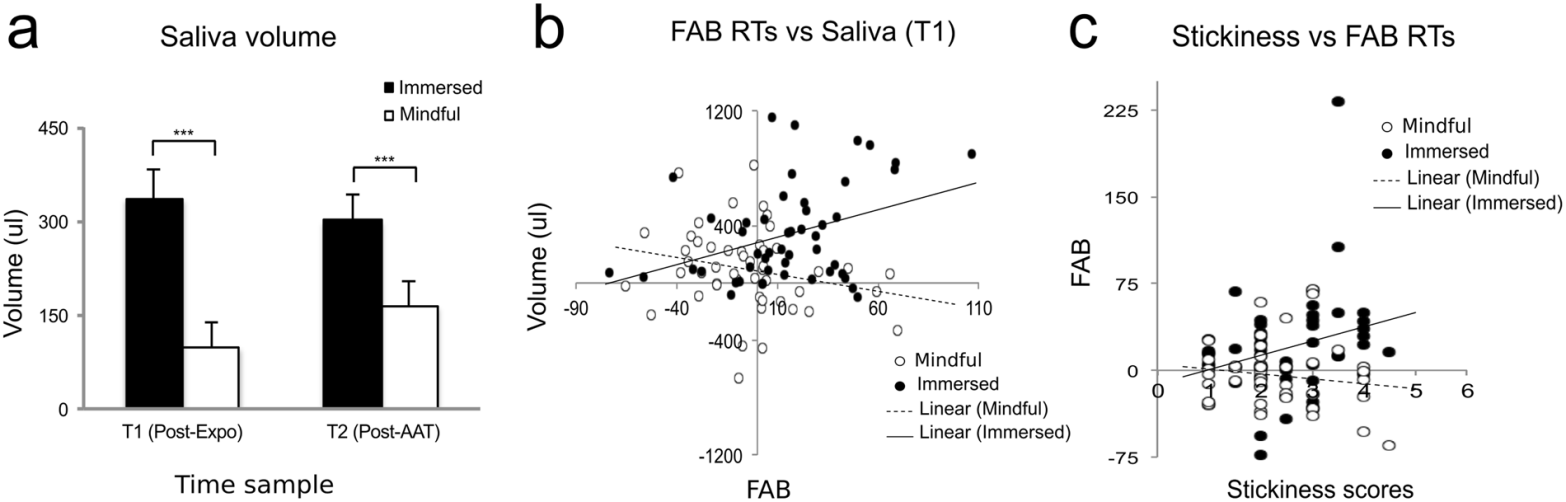
Integrative analysis of self-reports, behavioral and physiological measures: (**a**) Saliva volumes increased compared to baseline (T0) during the Immersed condition compared to the Mindful condition. (**b**) Saliva volume was predicted by FAB during the Immersed condition (for each ms on the FAB value, saliva volume increased by 0.01 ml), but not during the Mindful condition. (**c**) FAB RTs were predicted by the stickiness sub-scale during the Immersion instruction only (each point of stickiness scale predicts an increase of 12.32 ms on the FAB value). In all graphs, bars denote standard error. Significances *p < *0*.05, **p < *0*.01, ***p < *0*.001.

We evaluated whether, in line with the literature, the presentation of food cues during exposure triggered cephalic phase responses (CPRs) (i.e. increase in salivation). We then asked whether the Mindful attention instruction reduced CPRs during exposure to food cues when compared to the Immersion instruction. As T0 average volumes were not different between the two conditions (t-test *t*(49) = 0.09, *p* = 0.9), salivary volumes at T1 and T2 samples were normalized by regressing out volumes to baseline samples (T0) across both conditions. In line with our first prediction, salivary volumes showed an increase at T1 (*M* = 210; *SD* = 234, t-test, *t*(49) = 6.4, *p* < 0.001) and T2 (*M* = 232; *SD* = 222, t-test, *t*(49) = 7.4, *p* < 0.001). Next, we investigated the effect of Group and Instruction type over salivary volume fluctuation, by conducting a Group x Instruction type x Time sample (T1/T2) repeated-measure ANOVA. In line with our second prediction, we observed a main Instruction type effect (F(1, 48) = 16, p < 0.001, η^2^_G_ = 0.09) due to a higher increase in salivation during the Immersed condition (*M* = 318; *SD* = 262) compared to the Mindful attention condition (*M* = 131; *SD* = 270) (t-test, *t*(49) = 4, *p* < 0.01) (Fig. 2a). This finding demonstrates that the preparatory food intake reflex was down-regulated by the Mindful attention instruction. There was no other Group-related effect in this analysis.

### Salivary Alpha-Amylase

As Alpha-amylase is secreted in the presence of food as part of the CPRs, we hypothesized that an increase in Alpha-amylase activity would take place in response to food cues. We also expected that this effect would be more pronounced in the Immersed condition compared to the Mindful attention condition. In line with our first prediction, Alpha-amylase activity showed an increase at T1 (*M* = 30.5; *SD* = 51.5) (t-test, *t*(49) = 6.14, *p* < 0.001) peaking at T2 (*M* = 52; *SD* = 71) (t-test, *t*(49) = 7.9, *p* < 0.001), demonstrating the sensitivity of alpha-amylase activity to our experimental manipulation (Supp. Fig. 6). To explore Group and Instruction type effects over salivary alpha-amylase activity we conducted a Group x Instruction type x Time sample (T1/T2) repeated-measure ANOVA. We found a main effect of Time sample (F(1,48) = 4.9, *p* < 0.05, η^2^_G_ = 0.006) due to higher Alpha-amylase activity (U/mL) at T2 (*M* = 52; *SD* = 46.7) compared to T1 (*M* = 30.4; *SD* = 35, t-test, *t*(49) = 3.8, *p* < 0.01. This enhanced Alpha-amylase activity during the active part of the task (T2, AAT) compared to the passive task (T1, exposure) is in agreement with previous work^24^. Contrary to our hypothesis, however, Group or Instruction factors did not modulate this measure (for further discussion please see supplementary materials).

### State Questionnaires

We used an in-house questionnaire scale to determine whether the Instruction condition impacted self-report measures of subjective realism, and to confirm that subjects properly followed the instructions (see Supp. for details). Consistent with our manipulation, the global score of subjective realisms from the post-state questionnaires were significantly higher during the Immersed condition, (*M* = 11.5, *SD* = 2.2) compared to the Mindful attention condition (*M* = 9.9, *SD* = 1.6), (t-test, *t*(49) = 5.5, p < 0.001). (Supp. Fig. 5). Specifically, participants reported less *craving* during the Mindful condition compared to the Immersed condition (*M* = 3, *SD* = 0.87 and *M* = 3.44, *SD* = 0.9, respectively, t-test, *t*(49) = 5.5, *p* < 0.001), greater *meta-awareness* (resp. *dereification*) in the Mindful condition (*M* = 4.1, *SD* = 0.5, resp. *M* = 3.2, *SD* = 0.97) compared to the Immersed condition (*M* = 3.8, *SD* = 0.50, resp. *M* = 2.7, *SD* = 0.95) (*t*(49) = 3.5, *p* < 0.001, resp. *t*(49) = 4.1, p =  < 0.001) (Supp. Fig. 5b). *Stickiness*, on the other hand, was greater in the Immersed condition compared to the Mindful condition only at a trend level (*M* = 2.5, *SD* = 1, and *M* = 2.3, *SD* = 0.9 respectively),(t-test, *t*(49) = 1.9, *p* = 0.06, Supp. Fig. 5b).

Contrary to our hypothesis of a meditation-related effect during the mindfulness condition, there were no Group by State interactions between these variables (see Supp. for details).

### Relationship between Behavioral and Salivation Measures

We performed Pearson correlations, and a subsequent linear regression analysis, to investigate whether instruction-related changes on FAB predicted instruction-related changes in salivation. We hypothesized that greater FAB during AAT would predict greater salvation following the exposure phase (T1 sample).

In line with our prediction, we found that the increase in saliva volume following the exposure phase positively correlated with FAB values in the Immersed condition (r = 0.4, *p* < 0.01) but not in the Mindful condition (r = −0.002, p = 0.9), (Fig. 2b). To better describe the relationship between these two measures, we performed a multiple linear regression model analysis, predicting salivation volume from the FAB value (see Methods), where we found an overall fit to the model (Adjusted R^2^ = 0.2, F (3,94) = 8.8, *p* < 0.001). Specifically, there was a main effect of Instruction type (β = −198, t = −3, *p* < 0.01), whereby, in the Immersed condition, there was a greater increase in salivation when compared to the Mindful condition. We found a FAB value main effect (β = 3.8, t = 2.9, *p* < 0.01) implying that participants with greater FAB values salivated more. Importantly, there was also an interaction between Instruction type and FAB values in the prediction of saliva volume (β = −6.2, t = −3, *p* < 0.01), indicating that during the Immersed condition, for every ms of increase in the FAB value, salivation increased by 0.01 ml, while FAB values during Mindful condition did not predict salivation amounts. The difference in the correlational slopes between the two conditions indicates that salivary volumes were positively correlated with FAB values only in the Immersed condition, but not the Mindful condition.

### Relationship between Self-report and Behavioral Measures

We used a linear regression analysis to evaluate whether individuals that report greater differences in subjective realism between the Mindful and Immersed conditions during the exposure phase, (as measured by the overall score of the State questionnaire or by the individual scores from each of its four sub-scales separately), also exhibited higher food attractiveness bias in the AAT (as measured by FAB values).

We found that only the model for the *stickiness* sub-scale predicting FAB values was significant (Adjusted R^2^ = 0.1, F(3,96) = 5.6, *p* < 0.001). In this model, there was a main effect of *stickiness* scores (β = 0.01, t = 2.2, *p* < 0.05) where one point of increase in the sub-scale score predicted an increase of 12 ms in the FAB value. There was a *stickiness* score x Instruction type interaction (β = −0.016, t = −2.0, *p* < 0.05), indicating that correlational slopes during Mindful and Immersed conditions were significantly different: during the Immersed condition, one point of increase in the *stickiness* subscale score predicted an increase of 16.2 ms in the FAB values, while during the Mindful condition, *stickiness* scores did not predict the FAB value (Fig. 2c). In summary, among the four sub-processes that we explored, only the *stickiness* subscale predicted FAB values.

### Relationship between Trait and State Measures

To investigate whether changes in subjective realism in the AAT paradigm predicted relevant trait measures associated with mindful attention and immersion, we analyzed the relationship between condition-related measures (i.e. State questionnaire, FAB values and saliva volumes) and trait cognitive fusion, trait dereification, trait food craving measures and, for meditators, hours of meditation practice in their life. We corrected these analyses for multiple corrections (Bonferroni corrections, alpha = 0.016). We hypothesized that the more the participants were self-immersed in the food images, the more they would have a proclivity for food craving in daily life, and the higher they would score on cognitive fusion as a trait. Conversely the more the participants were successfully applying the mindful attention instruction, the higher they would score on dereification and, for meditators, have more meditation experience.

**Figure 3:**
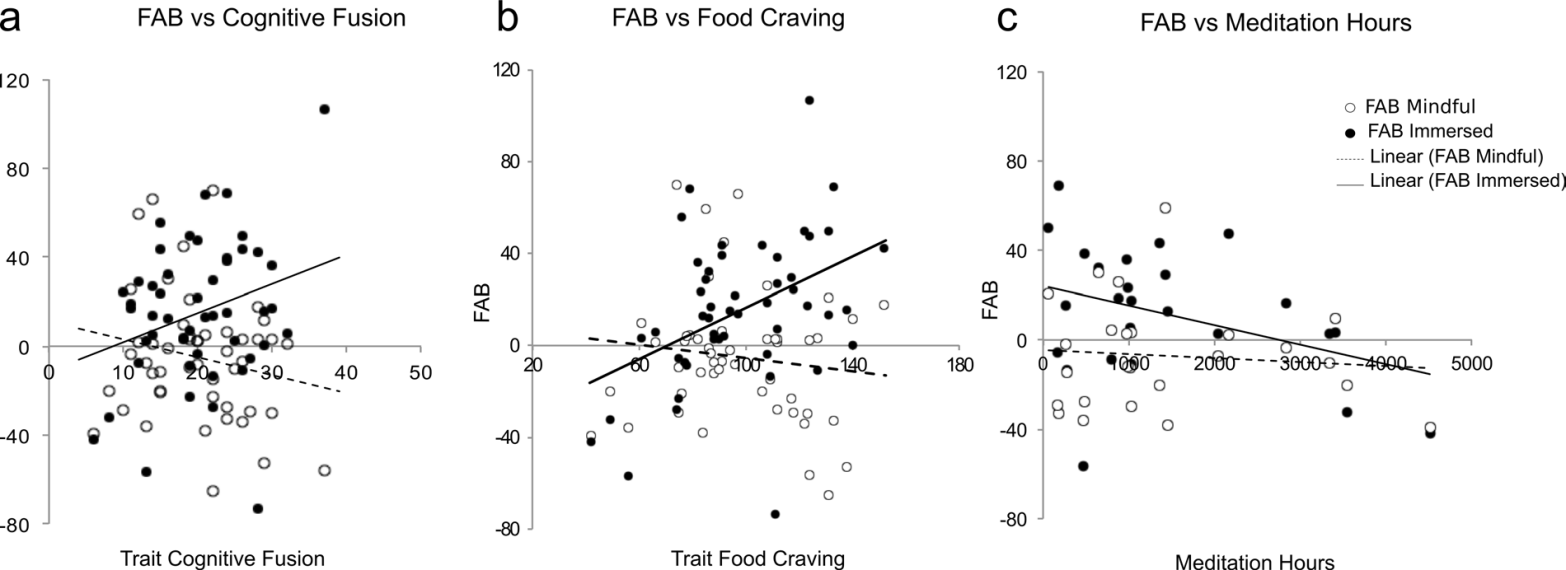
Trait-State integrative analyses: (**a**-**b**) Cognitive fusion (resp. Food craving) as a trait scores predicted FAB values during Immersed condition as measured by a linear regression model (each point of increase in the score from the Cognitive fusion questionnaire (resp. Food craving) corresponded to an increase in the FAB effect of 1.3 ms (resp. 0.55 ms) but not during Mindful condition). (**c**) Hours of lifetime meditation practice predicted FAB values only during immersed condition (for each 100 hours of meditation training there was a decrease in the FAB effect of 0.9 ms).

As predicted, scores in food craving as a trait positively correlated with FAB during the immersed condition (Pearson r = 0. 36, *p* < 0.01), but not during the mindful condition (Pearson r = −0. 12, *p* = 0.38) (Fig. 3b), indicating that participants who have a greater tendency toward food craving as a trait, also exhibited greater differences between RTs for avoidance and approach responses toward attractive food. These two measures were not correlated under the mindful condition, suggesting that the trait signature of food craving was offset by the mental state induced by the mindful attention instruction. To refine the analysis of the relationship between these two measures, we used food craving as a trait score and Instruction type to predict FAB in a multiple linear regression model. We found that the overall fit of the model was significant (Adjusted R^2^ = 0.17, F (3,94) = 7.8, *p* < 0.001). There was a main effect of Instruction type (β = 48.4, t = 2.0, *p* < 0.05) driven, again, by a higher FAB during the Immersed condition compared to the Mindful condition. There was also a main effect of food craving as a trait score (β = 0.56, t = 3.30, *p* = 0.001), whereby participants reporting higher food craving scores exhibited greater FAB values. Finally, we found an Instruction type x food craving interaction (β = −0.7, t = 2.0, *p* < 0.01), where a one point increase in the food craving questionnaire score was equivalent to an increase of 0.55 ms in the FAB value in the Immersed condition (Fig. 3b). In contrast, FAB was not correlated with trait food craving in the Mindful condition. We found a similar result with cognitive fusion as a trait (Adjusted R^2^ = 0.13, F (3,94) = 5.65, *p* < 0.001) (Fig. 3a). Dereification as a trait did not predict FAB, even if the overall model was significant (Adjusted R^2^ = 0.095, F (3,94) = 4.42, *p* < 0.005, see Supp. Table 1).

We also studied the relationship between FAB values and the total number of hours of meditation that the meditators had practiced in their lives. We found that the hours of life time meditation practice, were negatively correlated with immersed FAB values (Pearson r = −0. 36, *p* < 0.05) but not with mindful FAB values (Pearson r = −0.1, *p* = 0.6) (Fig. 3c). These results where not confirmed by a FAB Instruction interaction in the regression multiple analysis. This was most likely due to a lack of statistical power for the sample size used. For each 100 hours of meditation practice, there was a decrease in the FAB effect of 0.9 ms.

Finally, in their self-reports, meditators differed from controls on several trait and state measures concerning mindfulness, cognitive fusion, food-trait and decentering (Supp. Fig. 1). Some of these measures correlated with total number of practice hours. As the Group factor did not interact with the behavioral and physiological measures, we present these findings in the supplementary materials (Supp. Figs 1, 3, 5, and 8).

## Discussion

### Behavior

We found the same behavioral effects as the main results obtained by Papies *et al*. (2012) and extended them to a modified intra-subject protocol suitable for psychophysiological recordings and qualitative measures. Specifically, we showed a reduction of automatic approach bias toward attractive food (FAB) following a mindful attention instruction when compared to an immersion instruction. Similarly, in the field of psychotherapeutic and mindfulness-based interventions (MBI), decentering-related constructs akin to mindful attention have been identified as one of the core mechanisms to overcome maladaptive behavioral, cognitive or affective habits^2,7,9,25,26^. Examples of de-automatization mediated by mindfulness meditation have already been reported in attention, perception and physiological reflex^27–29^.

### Autonomic physiology

We collected participants’ salivation profiles under each instruction to characterize an objective physiological measure of subjective realism toward food cues. Although increase in salivation in response to food cue exposure or food imagery is a well-known phenomenon^11,30,31^, its regulation by a mindful attention instruction has not been shown before. Here we observed a reduction in saliva volume during the mindful attention instruction when compared to the immersion instruction. We also found that, during the immersed condition only, salivation correlated positively with the FAB value: participants who salivated more during exposure to food images exhibited higher FAB values in the AAT, which indicates stronger impulses toward attractive food. In cognitive terms, self-immersing in the food cue could be considered to simulate eating or tasting the food displayed by the cue. This mechanism would enhance the intake preparation reflex, strengthening the encoding of the food cue in memory and, therefore, leading to greater impulsive approach reactions toward the attractive food during the AAT. In trials where the required action was opposed to the automatic tendency (i.e. avoid attractive food), the first automatic impulse would be more difficult to overcome in the context of an enhanced intake preparation reflex, thus resulting in greater FAB values.

By contrast, during the mindful attention condition, there was a reduction in both saliva volume and the FAB. In this condition, it was not possible to establish a relationship between salivation and FAB values in the AAT performance. These findings could be accounted for by either weaker eating simulation and autonomic preparatory food intake reflex, related to a different encoding of food images^32,33^, or by a stronger regulation of executive conflict during the AAT performance, a capacity known to be affected by mindful attitude and training^34,35^. Accordingly, the smaller increase in saliva volume found during the exposure phase is consistent with an encoding regulation hypothesis, even though the absence of the FAB effect during the mindful condition could also be partially accounted for by an enhanced response inhibition mechanism. An examination of the brain mechanisms underlying behavior in this paradigm is needed to settle this issue.

### Self reports

The results of the post-state questionnaire indicated that participants were able to identify and differentiate their feelings, reporting less craving and greater meta-awareness and dereification in the mindful condition when compared to the immersed condition. Stickiness scores were higher for the immersed condition compared to mindful attention, albeit at trend level. Only the stickiness sub-scale predicted the FAB effect. From these results, the stickiness construct appears to be the most promising dimension to predict AAT behaviors. Contrary to our prediction, the two sub-processes of mindful attention, namely, meta-awareness and dereification, did not predict FAB. Further work is needed to identify the specific contribution of each of these sub-scales, in particular using more refined first-person interviews^36^.

### Trait

The food craving-as-a-trait questionnaire was able to predict the FAB effect. This correlation disappeared during the mindful condition, suggesting that this “food craving as a trait signature” is offset by the mental setting provided by the mindful attention instruction, as expressed by the AAT RTs. We observed a comparable finding for cognitive fusion as a trait, but not for dereification as a trait.

Scores from the food craving-as-a-trait questionnaire were positively correlated with BMI, which was expected, and consistent with the literature^37^. Participants with greater trait cognitive fusion scores also had greater trait food craving scores, indicating that these two constructs are related as trait features. Interestingly, trait food craving was negatively correlated with the dereification-as-a-trait questionnaire scores, highlighting the relationship between automatic tendencies toward food and trait subjective realism also revealed by the AAT performance. To our knowledge, this relationship has not been reported in the literature before. From these correlations, high cognitive fusion/low derefication as a trait appears as a broader feature that might be consistent with stronger food cravings, hence facilitating greater BMIs as a result. This finding is not surprising since excessive experiential fusion is a hallmark of psychological distress^38^, depression^39^, and addictive disorders^40^, which are the most common co-morbidity features in overweight and obesity conditions.

### Meditation expertise

Consistent with previous studies, meditators exhibited higher scores in the five-facet mindfulness questionnaire^41^. Compared to non-meditators, they scored higher in the in-house dereification-as-a-trait questionnaire, and lower in the cognitive fusion questionnaires. Meditators scored lower on a food craving questionnaire at a trend level, and this finding is consistent with recent results by Papies *et al*.^42^. Importantly, the total number of practice hours was positively correlated with the scores of the five-facet mindfulness questionnaire, negatively correlated with cognitive fusion and food craving-as-a-trait questionnaire, and negatively correlated with the AAT FAB effects. The relationship between meditation practice and trait measures that are relevant for food disorders should be further tested using a more appropriate longitudinal randomized controlled trial design.

We initially hypothesized that meditation experience would facilitate mindful attention during the instruction. Despite finding main Group effects on both behavioral results and state self-report scores, we found no interaction with Group (Supp. Figs 3 and 5b). Specifically for behaviors, the lack of 4-way interaction indicated that the capacity to sustain mindful attention toward food cues, as requested by a brief instruction, does not require formal training to induce detectable behavioral effects, and that meditation experience does not influence the capacity of self-immersing. Therefore, to differentiate the meditators from controls, a non-instructed condition would have been more appropriate to reveal any possible predisposition to engage spontaneously in mindful attention, which would result from long-term mindfulness practice. As discussed below, this additional condition was not included in our paradigm.

### Limitations

We did not include an uninstructed control task, so it is difficult to separate the effect of immersion and mindful attention specifically. Including such condition would have made the task too lengthy and therefore susceptible to participant’s fatigue. However, previous studies have already established a reduced effect of mindful attention compared to an uninstructed condition on AAT behaviors^8^, and an enhancement of saliva volume in an immersed condition compared to uninstructed condition^43^. Here we chose to maximize the changes in subjective realism to study its neuro-electrical correlates. Despite this, in our approach, we were still able to investigate the specific contribution of immersion and mindful attention using within-subject variability (see Figs 2–3).

### Future directions

Contrary to our expectation, the functional relationships between our dependent measures and state and trait self-report measures were statistically significant only during the immersed condition rather than during the mindful condition. The analysis of electrophysiological data and qualitative reports from mindful attention instruction collected during this study seem to be promising for further understanding of the neural and cognitive mechanisms of this regulation.

## Electronic supplementary material

Supplementary Material
