# Supplementary material for: Compared to self-immersion, mindful attention reduces salivation and automatic food bias
Source: Sci Rep. 2017 Oct 23;7:13839. doi: 10.1038/s41598-017-13662-z (PMC5653876; doi:10.1038/s41598-017-13662-z)
Supplement: Supplementary file 1 — Supplementary Material [file 41598_2017_13662_MOESM1_ESM.pdf]

SUPPLEMENTARY MATERIAL

# Compared to self-immersion, mindful attention reduces salivation and automatic food bias.

Constanza Baquedano [a,b,c], RodrigoVergara [d], Vladimir Lopez [b,c,d], Catalina Fabar [b], Diego Cosmelli [b,c,d], Antoine Lutz [a].

Table of Contents

Table of Contents 2
1) Supplementary Methods: 3
1.1) Meditator Group Validation: 3
1.2) In-house Questionnaire Validations: 4
1.3) Results of the Questionnaire Validations: 4
1.4) Display In-house Questionnaires: 5
Pre-State Questionnaire: 5
Post-State Questionnaires: 6
Trait Questionnaire 9
1.5) Display Instructions: 12
Textual Adapted Instructions: 12
2) Supplementary Results: 13
2.1) State Measures: 13
2.1.1) Pre-State Questionnaire 13
2.1.2) Reaction Times by Group: 13
2.1.3) Order Effect of Instruction on Behavior: 15
2.1.4) Post-State Questionnaire: 16
2.1.5) Alpha-Amylase results: 17
2.2) State and Trait Integrative Analyses: 19
2.2.1) FAB in Relation to Dereification as a Trait Questionnaire: 19
2.3) Trait Measures: 19
2.3.1) Trait Integrative Analysis: 19
2.3.2) Trait Integrative Analysis by Group: 20
References 22

1) Supplementary Methods:

1.1) Meditator Group Validation:

We used questionnaires described in the Methods section to characterize the meditator group compared to the control group. We also investigated the relationship between relevant trait measures such as food craving and dereification with the total number of hours of meditation practice. These analyses are summarized in Supplementary figure 1.

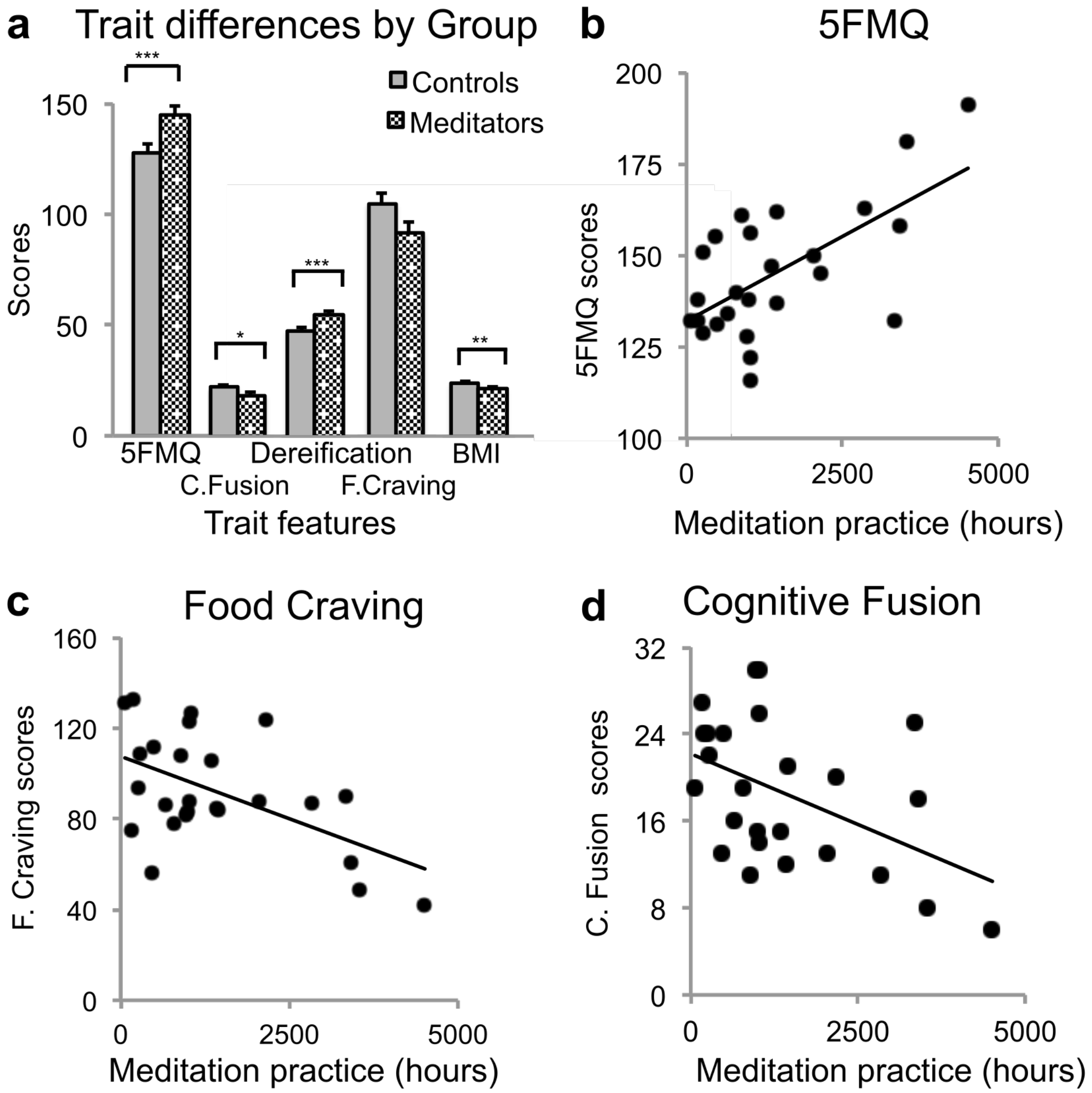

Supplementary figure 1, Trait meditation experience analysis: a) Meditators presented higher scores in the Five-Facet Mindfulness Questionnaire, a standard measure of trait mindfulness[1,2], (Med, M=145.2 SD=17.8; Cont., M=127.9 SD=19.4) (t-test, $t(24)=4.1$, $p<0.01$), higher scores in our in-house Dereification

Questionnaire (Med, M=34.7 SD=5.3; Cont., M=29.4 SD=4.7) (t-test, $t(24)=4.1$, $p<0.01$), and lower scores in the Cognitive Fusion Questionnaire (Med, M=18.5 SD=6.7; Cont., M=22.3 SD=7.06) (t-test, $t(24)=2.1$, $p<0.05$). Food craving as a trait scores were lower for meditators at a trend level (Med, M=92 SD=25; Cont., M=105 SD=24.4) (t-test, $t(24)=2$, p=0.058). Meditators presented lower BMI than non-meditators (Med, M=21.9 SD=2.7; Cont., M=24.2 SD=2.6) (t-test, $t(24)=2.9$, $p<0.05$). **b)** The scores of the Five-Facet Mindfulness Questionnaire were positively correlated to total number of practice hours (r = 0.6, $p<0.05$), **c),** negatively correlated with the scores of Food-Craving Trait Questionnaire (r = -0.54 , $p<0.01$), and with the score of the Cognitive Fusion Questionnaire (r = -0.5, $p<0.05$). Bars denote standard errors. Significances *$p<0.05$, **$p<0.01$, ***$p<0.001$.

1.2) In-house Questionnaire Validations:

We applied a confirmatory factor analysis to our in-house Dereification-as-a-Trait and Post-State questionnaires by evaluating the robustness and orthogonality of these constructs. We used a Maximum Likelihood Factor extraction method[3], and then rotated these factors by using an Oblique Oblimin Rotation method [4]. We extracted as many factors as the number of constructs that we proposed in each questionnaire. As presented below, we confirmed that the number of factors was consistent with the number of constructs for each questionnaire, and that each scale and sub-scale/construct was internally consistent.

1.3) Results of the Questionnaire Validations:

Trait Questionnaire: The Trait Dereification Questionnaire was subject to a factor analysis and internal consistency analysis. Factor analysis, using parallel analysis, optimal coordinates, and acceleration factor as extraction methods, suggested the extraction of two factors. However, this solution was not significant (χ^2=101.2, df=89, p=0.18). Since the original hypothesis considered one factor, factor analysis was re-run extracting only one factor. Items with factor loadings under 0.35 were dropped, leading to a questionnaire of ten questions. These ten items, extracted as one factor, were significant (χ^2=74.2, df=35, p<0.0002) and accounted for 35% of the variance. Finally, internal consistency was assessed, presenting a Cronbach's Alpha of 0.83. The validated Trait Dereification Questionnaire presented a significant correlation with the Cognitive Fusion Questionnaire (t = -9.3, df = 48, p < 1e-11) with an R^2 of -0.80.

State Questionnaire: We used the eigenvalues criteria for factor extraction, which fitted the original hypothesis of four factors (sub-scales). No cross loading was detected and only one item was dropped due to factor loading under 0.35. The cumulative variance explained by the four factors was of 54%, and the number of factor extracted was sufficient (χ^2=121.4, df=74, $p<0.001$). The Cronbach's Alpha of each sub-scale was 0.93 for Dereification, 0.84 for Craving, 0.84 for Stickiness, and 0.55 for Meta-awareness. We found that our sub-scales were not orthogonal, evidenced in the significant correlations between Dereification with Craving ($r = -0.6$, $p<0.001$), Dereification with Stickiness ($r = -0.32$, $p<0.01$), Meta-awareness with Craving ($r = 0.3$, $p<0.005$), and Craving with Stickiness ($r = 0.4$, $p<0.001$). These correlations highlight the relationships between Dereification and Meta-awareness and Craving and Stickiness.

In the *Dereification* sub-scale, we attempted to determine how strongly people considered their perceptions and sensations of food images to be real, and how strongly these reified perceptions triggered bodily sensations *(*e.g*. How vividly did the food images evoke feelings in you? How "real" did they feel?)*. In the *Meta-awareness* sub-scale, we assessed how aware participants were of their thoughts, and how much cognitive distance they had with them (e.g*. During the food image presentation I could relate to my thoughts as mere mental events that came and went)*. With the Craving sub-scale, we measured how much food craving was induced during the paradigm (e.g. *While I was watching the images of food I felt desire to eat the food)*. With the Stickiness sub-scale, we evaluated how difficult it was for the subjects to overcome or "let go" the food-related thought at the end of a trial (e.g.*: In the passage from one image to another, I usually kept thinking about the previous one*).

1.4) Display In-house Questionnaires:

Pre-State Questionnaire:

Instructions: In the next section, please answer from 1 to 5 the option which best reflects your experience at the moment. With 1 being not at all and 5 very much

1) Are you happy right now?

¿Te sientes contento en este momento?

2) Are you hungry right now?

¿Estas hambriento en este momento?

3 Do you feel anxious right now?

¿Te sientes ansioso en este momento?

4) Do you want to eat?

¿Tienes deseos de comer?

Post-State Questionnaires:

**Instruction:**<br>From item 1 to 12, the instructions are as follows: Using the 1-5 scale shown below, please indicate the extent to which you agree or disagree with the following statement regarding your experience during the task:<br>1- strongly disagree, 2-disagree 3- neither agree nor disagree 4- agree, 5- strongly agree.<br><br>From item 12 to 20, the instructions are the following: Using the 1-5 scale below, please indicate the statement which most accurately represents your experience at the moment.<br>1-not represented at all and 5- Accurately represented
Reality-Dereification items: 9 13 14 15 16 17 20
9) The sensations that I experienced from my thoughts about the food images were very similar to those I experience when I am in front of real food.<br>*(Las sensaciones que experimenté a partir de mis pensamientos sobre las imágenes de comida eran muy similares a las sensaciones que experimento cuando estoy frente a los ítems de comida reales.)*
13) How intensely did you feel the sensations evoked by the food images?<br>*(¿Qué tan intensas sentiste las sensaciones evocadas por las imágenes de comida?)*
14) How emotionally involved were you with the feelings evoked by the food images?<br>*¿Qué tan involucrado emocionalmente te sentiste con las sensaciones evocadas por las imágenes de comida?*
15) How vivid were the food items for you? (How "real” did they feel?)<br>*¿Cuán vividos te parecieron los ítems de comida? (que tan “real” los sentías)*

16) How immersed in the food images were you?<br>*¿Qué tan inmerso te sentiste en las imágenes de comida?*
17) How immersed were you in the feelings evoked by the food images?<br>*¿Qué tan inmerso te sentiste en las sensaciones que te evocaron las imágenes de comida?*
20) How vivid were your feelings during the perception of the food images? (How "real" did they feel?)<br>¿Cuán vividas eran tus sensaciones evocadas por las imágenes de comida? (que tan "real" se sentían)
Craving: 1 2 6 7 18
1) As the images were appearing, my appetite increased<br>*A medida que iban apareciendo las imágenes aumentaba mi apetito*
2) As the images were appearing, my appetite decreased<br>*A medida que iban apareciendo las imágenes disminuía mi apetito.*
6) While I was looking at the food images, I felt desire to eat them.<br>*Mientras veía las imágenes de comida me daban ganas o deseos de comerlas.*
7) While I was looking at the images "my mouth watered." ("mouthwatering")<br>*Mientras veía las imágenes se me hacia agua la boca.*
Stickiness: 4 5 10
10) While I was looking at the food pictures I was usually "dwelling in" my thoughts of these images.<br>*Mientras veía las imágenes de comida, los pensamientos respecto a estas se mantenían "dando vueltas" en mi mente,*
4) In the transition from one image to another, I usually remained thinking about the previous one.<br>*Cuando pasaba de una imagen a otra, generalmente me quedaba pensando en la imagen anterior*
5) In the transition from one image to another, the sensations triggered by the previous one remained with me<br>*Cuando pasaba de una imagen a otra, generalmente mis sensaciones sobre la primera imagen permanecían.*
Meta-awareness: 3 8
3) During the food image presentation I could relate to my thoughts as mere mental events which came and went.<br>*Durante la presentación de las imágenes pude relacionarme con mis pensamientos sobre estas, como meros eventos mentales que iban y venían.*

8) While I was looking at the pictures, I realized that the thoughts, sensations, or feelings about them were triggered by my subjective interpretations of them, and that they did not necessarily correspond to qualities contained in the images themselves (and because of this other people could have thoughts and feelings toward the same item different from mine).<br>*Mientras veía las imágenes me daba cuenta de que los pensamientos y sensaciones respecto a ellas eran mas bien interpretaciones subjetivas mías de las imágenes y no necesariamente correspondían a cualidades contenidas en ellas (y por esto, otras personas podrían tener otros tipos de pensamientos y sensaciones diferentes a las mías frente un mismo ítem de comida).*
NON-SELECTED ITEMS
12) While I was looking at the food pictures I usually realized the contents of my thoughts about them.<br>*Mientras veía las imágenes generalmente me daba cuenta del contenido de mis pensamientos respecto a ellas.*
18) How intensely did you feel your impulses of wanting to eat the attractive food items?<br>*¿Qué tan intensos sentiste tus impulsos de querer comer los ítems de comida ricos que aparecían en las imágenes?*
19) How intensely did you feel your impulses of wanting to eat the NON-attractive food items?<br>*¿Qué tan intensos sentiste tus impulsos de querer comer los ítems de comida NO ricos que aparecían en las imágenes?*

Trait Questionnaire

Instructions: Each question below is followed by a number of response options. After reading each question carefully, choose the option that applies to you most. Read each question carefully and enclose in a circle the number that best describes the answer. (There are no correct or incorrect answers). - Never - Very seldom - Sometimes - Often - Very often<br>*Cada pregunta a continuación es seguido por un número de opciones de respuesta. Después de leer cada pregunta con cuidado, elija la opción que más se aplica a usted. Lea cada una cuidadosamente y encierre en un circulo el número que mejor lo describe, en general. (No existen las respuesta correctas o incorrectas). - nunca -muy rara vez -A veces -A menudo -Muy a menudo*
**Questions:**
1) I tend to relate to my thoughts as mere mental events that come and go.<br>*Tiendo a relacionarme con mis pensamientos como meros eventos mentales que vienen y se van.*
2) I tend to stay "spinning" in my thoughts.<br>*Tiendo a quedarme "dándole vueltas" a mis pensamientos.*
3) My thoughts produce all kinds of emotional and bodily sensations.<br>For example, if I think of some food, it immediately makes me want to eat, or if I think of a difficult situation that I have had in the day or that I will have later, it gives me an emotional feeling of anguish accompanied by the body sensation associated with the anguish (my gut or throat tightens)<br>*Mis pensamientos me producen todo tipo de sensaciones emocionales y corporales.*<br>*Por ejemplo, si pienso en algo de comida enseguida me dan ganas de comer, o si pienso en una situación difícil por la que he pasado en el día o que voy a pasar más tarde, me da una sensación emocional de angustia acompañada de la sensación corporal asociada a la angustia (se me aprieta la guata o la garganta)*
4) When I think, I realize that these thoughts are rather my own subjective interpretation of a fact and do not necessarily correspond to the fact itself.<br>*Mientras pienso me doy cuenta de que esos pensamientos son mas bien interpretaciones subjetivas mías de un hecho y no necesariamente corresponden al hecho en si.*

5) When I contemplate the nature of my thoughts in response to an event, I generally realize that they are experiential in nature, and not necessarily reflecting the fact itself. (For example, in the situation of having to make a public presentation, at the very moment that I have to plug my pen drive into the computer, I doubt for a moment that I have the pen drive with me and I imagine that I left it at home. A tense bodily sensation of anguish immediately invades my body and a great confusion arises in my mind. After realizing that my pen drive was actually in my pocket, I can return to a state of tranquility similar to the one I had before these thoughts invaded my mind).<br>*Mientras pienso, generalmente me doy cuenta de que las cosas que experimento a partir de mis pensamientos, son más bien experiencias generadas por mis pensamientos respecto a el hecho y no necesariamente por el hecho en sí mismo. (Por ejemplo en la situación de tener que exponer frente a colegas, al momento que debo colocar mi pendrive en el computador del presentador, por un momento dudo si tengo el pendrive conmigo y pienso que se me quedo en la casa. Inmediatamente una sensación de angustia y adrenalina invade mi estado corporal y una gran confusión se sitúa en mi mente. Al corroborar que mi pendrive está en mi bolsillo, vuelvo a un estado de tranquilidad similar al que estaba antes de que este pensamiento se situara en mi mente).*
6) The emotions I experience from my thoughts on a particular situation are exactly the same as the emotions that I experience from that situation at the moment I am living it.<br>*Las emociones que experimento a partir de mis pensamientos sobre una situación en particular sonexactamente iguales a las emociones que experimento a partir de esa situación en el momento en que la estoy viviendo.*
7) I can become aware that a bodily sensation that I am experiencing is triggered by discursive thinking. (For example, I realize that my back is tense and then I understand that it is because I have had a stressful day, or I realize that I have a tight gut, because I have an exam in the afternoon).<br>*Cuando siento mi cuerpo, me puedo dar cuenta de que un estado corporal que estoy viviendo, está dado por mis pensamientos.*<br>*(Por ejemplo, me doy cuenta que tengo mi espalda tensa y luego entiendo de que es porque he pasado un día estresante, o me doy cuenta que tengo la guata apretada, porque debo hacer un examen en la tarde.*
8) I am aware that thoughts arise in my mind spontaneously.<br>*Tengo la sensación de que pensamientos se sitúan en mi mente sin que yo haya decidido pensarlos (sin mi intención)*

9) When I reflect on the things that I do not like or when I have the feeling that it is not good for me to think about them, it is difficult for me to pull my attention away from these thoughts.<br>*Pienso en cosas que no me gustan o tengo la sensación de que no me hace bien pensarlas,me es difícil sacar mi atención de esos pensamientos.*
10) When I think about a situation, I usually realize that this is "my vision" of the situation and that there may be more interpretations of that situation in other people's points of view.<br>*Cuando pienso sobre una situación, generalmente me doy cuenta de que esa es "mi visión" de la situación y que posiblemente existen mas interpretaciones sobre esa situación en la "visión"de otras personas.*
11) My thoughts make me anxious.<br>*Mis pensamientos me ponen ansioso.*
12) My thoughts sometimes reassure me.<br>*Mis pensamientos s veces me tranquilizan.*
13) My thoughts often make me euphoric.<br>*Mis pensamientos muchas veces me ponen eufórico.*
14) My thoughts make me angry.<br>*Mis pensamientos me ponen rabioso.*
15) When I have a thought, I can choose to relate to it as a simple mental event, which does not truly reflect reality.<br>*Cuando tengo un pensamiento, puedo elegir relacionarse con el como un simple acontecimientos mental, que no reflejan verdaderamente la realidad.*
16) When I have a thought, I can be aware of the emotions and bodily sensations that are induced by this thought.<br>*Cuando tengo un pensamiento, puedo ser consciente de las emociones y sensaciones corporales que se inducen por este pensamiento.*

1.5) Display Instructions:

Textual Adapted Instructions:

Based on previous pilot information, we used slightly different instructions than those in Papies et al. (2012) in order to clarify them, and to reduce the variability of cognitive strategies utilized by participants. ***Adapted Mindful Attention Instructions:*** *"Below you'll see a series of images, in response to which you will probably experience all kinds of thoughts, sensations and reactions. In this task, for each picture we ask you to be aware of your thoughts, sensations and reactions, and experience them just as mere constructions of the mind, which appear and disappear. For this purpose, it may help you take the following steps: Every time an image appears; 1) Be aware of your thoughts and reactions caused by the images, 2) Consider these thoughts as mere constructions of your mind, 3) Note these thoughts and sensations appearing and disappearing".* ***Adapted Immersed Instructions:*** *"Below you'll see a series of images, to which you will probably experience all kinds of thoughts and reactions. In this task, we ask you to try to immerse intensely with the sensations generated by each picture. For this purpose, it may help you to take the following steps: Every time an image appears; 1) Experience your feelings and thoughts about these as if they were real. 2) Immerse yourself completely in your feelings and thoughts about them, 3) Remain in this state of immersion while the image is present.*

2) Supplementary Results:

2.1) State Measures:

2.1.1) Pre-State Questionnaire

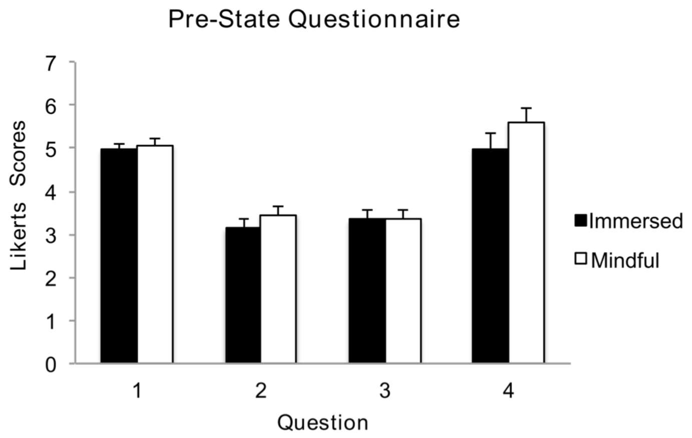

Supplementary figure 2: Liker scores on the four questions from the Pre-State Questionnaire. To discard the influence of any mood or hunger variables over the effect or the Instruction condition, we perform a 2-group X 2 condition repeated measure ANOVA to each of the four questions, i.e. 1) Are you hungry right now?; 2) Are you happy right now?; 3) Do you feel anxious right now?; 4) Do you want to eat?). None of these ANOVAs revealed any group or instruction effect. As a result of this, the modulation found during Instruction manipulation over RTs, saliva or self-reports, was not due to differences in any of those mood or hunger variables prior to condition block performance. Bars denote standard error.

2.1.2) Reaction Times by Group:

We analyzed the effects of Instruction (mindful attention vs. immersed), food type (attractive vs. neutral) and response type (approach vs. avoid) over RTs during the AAT using a 2 condition x 2 food type x 2 response repeated-measures ANOVA for each group separately. Supplementary figure 2 summarizes this analysis.

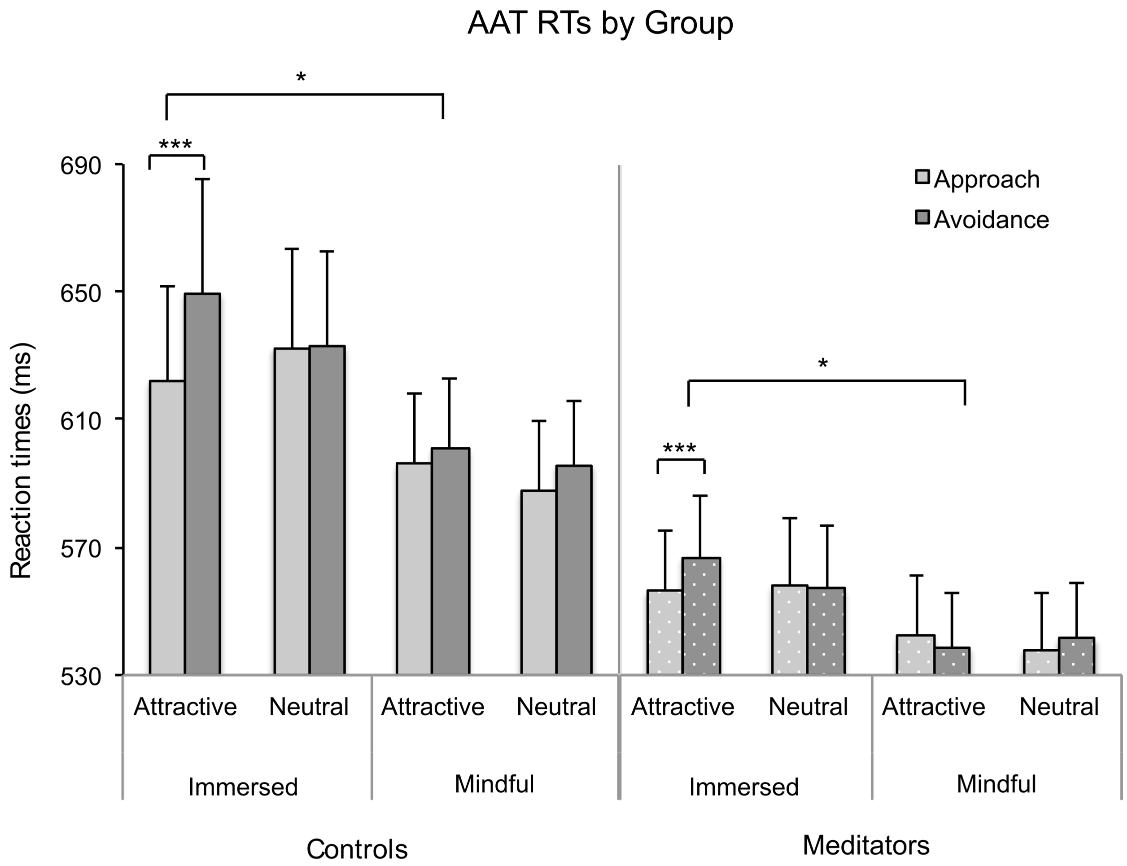

Supplementary figure 3. Reaction times (in ms) for approach and avoidance responses toward attractive and neutral food pictures, under both instructions. **(Left) Control group:** in the 2 Instruction condition x 2 Food type x 2 Response type ANOVA, we found a main Response type effect (F (1, 24) =9.6, p<0.01, η^2_G = .001), where approach RTs were faster than avoidance RTs. We found a Food type x Response type interaction (F (1, 24) =4.6, p<0.05, η^2_G = .0004) driven by slower RTs during avoidance compared to approach responses (t-test, t(24)=3.3, p<0.01), specifically for attractive compared to neutral food images (t-test, t(24)=1.4 p=.2. Consistent with our first hypothesis, we found an Instruction condition x Food type x Response type, F (1, 24) =4.5, p<0.05, η^2_G = .0008).

To further assess the three-way interaction, we examined the effects of Food type and Response type in the immersed and mindful attention conditions separately. In the immersed condition, there was a Response type effect (F (1, 23)= 8.9, p<0.05, η^2_G = .002) where approach RTs were faster than avoidance RTs (Table 1,). And we found a positive food attractiveness bias (FAB) in concordance with an interaction of food type and response type (F (1, 24)= 5.9, p<0.05, η^2_G = .001). This interaction was driven by faster approach responses than avoidance responses toward attractive food images (t-test, t(24)=3.3 p<0.01) that did not occur for neutral food images, (t-test, t(24)=.2 p=.8). By contrast, there was no FAB in the mindful condition, in

agreement with the lack of Food type X Response type interaction (F (1, 24)= .006, p=.6 , η^2_G = .00008). **(Right) Meditator group**: in the 2 Instruction x 2 Food type x 2 Response type repeated-measures ANOVA , we found an Instruction x Food type x Response type, F (1, 24) =8.9, $p<0.05$, η^2_G = .0006).

As before to further assess the last three-way interaction, we examined separately the effects of Food type and Response type in Immersed and Mindful attention conditions. In the Immersed condition, the 2 way interaction of Food type and Response type (F (1, 24)= 4, p=0.057, η^2_G = .0009) was present only as a trend. In the Mindful attention instruction there was no FAB effect, in concordance with the lack of Food type X Response type interaction (F (1, 24)=2.6, p=0.15. η^2_G = .0004). Bars denote standard error. Significances **p < 0.05*, ***p < 0.01*, ****p < 0.001*.

2.1.3) Order Effect of Instruction on Behavior:

To assess whether there was an order effect of the instructions on the behavioral performance, we performed a repeated measure ANOVA over RT with Group, Start-Instruction and Instruction as factors. This ANOVA did not reveal any Start-Instruction interaction (Instruction x Start-Instruction, F (1, 48) =0.18, p=0.7); Group x Instruction x Start-Instruction, F (1, 48) =2.2, p=0.2). The order effect of instruction did not introduce a confounder on the behavioral findings.

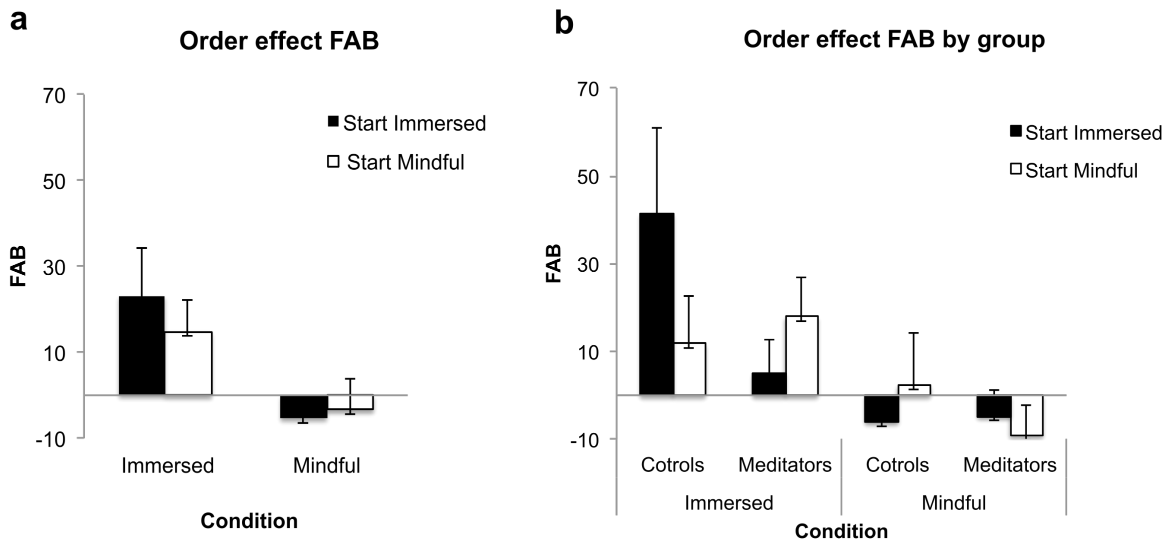

Supplementary figure 4: Order effect assessment **a)** FAB values for the Instruction x Start Instruction, F (1, 48) =0.19, p=0.7; **b)** FAB values for the Group x Instruction x Start-Instruction, F (1, 48) =2.2, p=0.1.

2.1.4) Post-State Questionnaire:

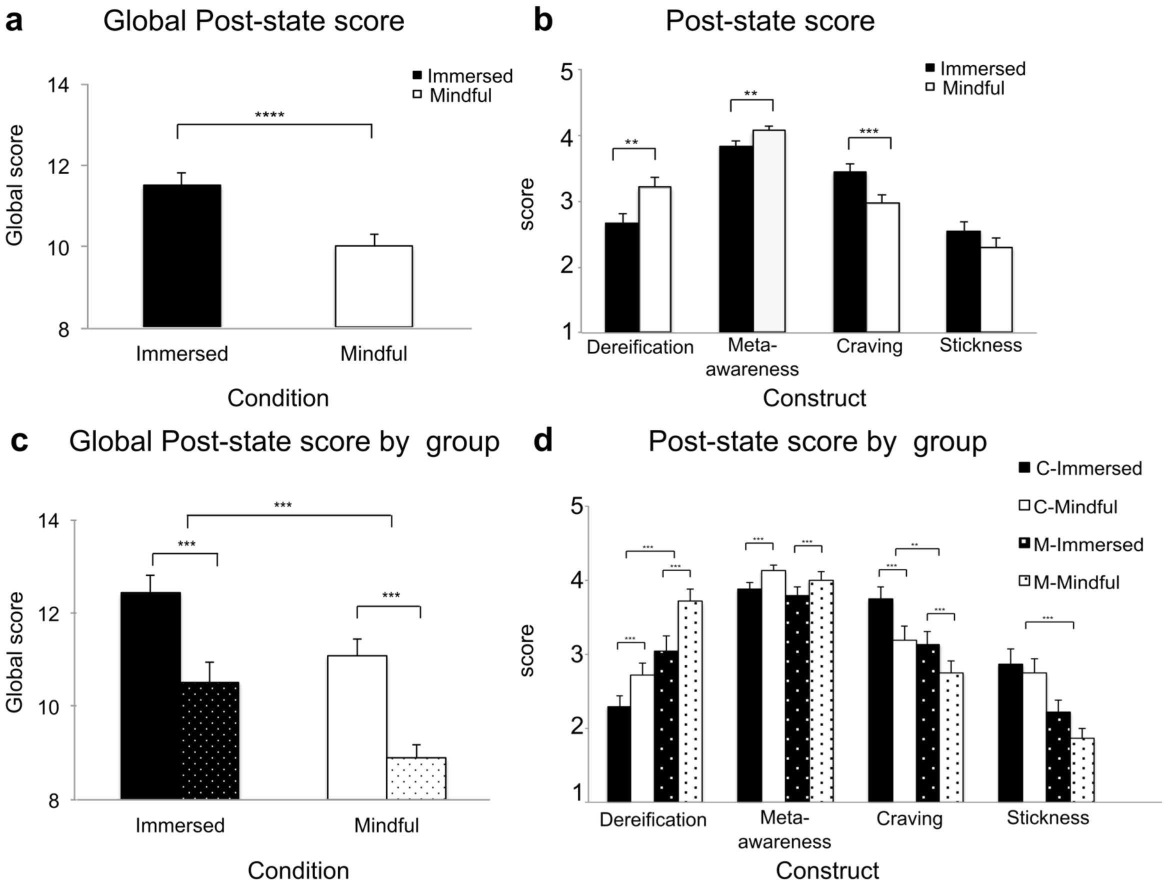

Supplementary figure 5: a) Global post-state scores following Mindful attention and Immersed conditions. Immersed scores (*M*=11.5 *SD*=2.2) were higher than Mindful attention scores (*M*=9.9 *SD*=1.6) (t-test, $t(49)=5.5$, $p < 0.001$). **b) Scores from each sub-scale of the Post-State Questionnaires after Mindful and Immersed conditions.** See description in the results section. **c) Global post-state scores following Mindful attention and Immersed conditions separated by group:** to assess the influence of previous meditation practice on global self-perceived subjective realism we performed a 2 Group x 2 Instruction condition repeated-measure ANOVA. In this analysis we found a main Group effect (F (1, 48)= 22.12, $p <$ 0.001. η^2_G = .24), where controls (*M*=11.8 *SD*=1.9) generally had greater scores of subjective realism compared to Meditators (*M*=9.7 *SD*=1.95). Also, there was a main Condition effect (F (1, 48)= 29.8, $p <$ 0.001. η^2_G = 0.2), where the Immersed condition presented higher scores (*M*=11.5 *SD*=2.1) than the Mindful attention condition (*M*=9.9 *SD*=1.6). **d) Scores from each sub-scale of the Post-State Questionnaires after Mindful and Immersed conditions separated by groups.** Next we performed a 2 Group x 2 Instruction condition repeated-measures ANOVA for each sub-scale. In the ***Dereification scale,*** there was a main Group

effect (F (1, 48)= 17.90, $p < 0.001$. η^2_G = .21) where meditators presented greater dereification scores (M=3.4, *SD*=0.99) than controls (*M*=2.5, *SD*=0.8). There was also a main Condition effect (F (1, 48)= 16.8, p= $p <$ 0.001. η^2_G = 0.1). Dereification scores during the Mindful attention condition (*M*=3.21 *SD*=1) were higher than in the Immersed condition (*M*=2.7 *SD*=1). In the ***Meta-awareness scale,*** there was a main Instruction condition effect (F (1, 48)= 11.7, $p < 0.01$. η^2_G = 0.05), where Meta-awareness scores were higher during the Mindful attention condition (M=4 SD=0.5) compared to the Immersed condition (*M*=3.8 *SD*=0.5). For the ***Craving scale,*** we found a main Group effect (F (1, 48)= 6.7, $p < 0.05$. η^2_G = 0.08), where controls had (*M*=3.5 *SD*=0.9) higher craving scores than meditators (*M*=2.9 *SD*=0.9), also there was a main Instruction condition effect (F (1, 48)= 13.2, p<0.001. η^2_G = 0.07), where the Immersed (*M*=3.4 *SD*=0.9) condition presented higher scores than the Mindful condition (*M*=2.9 *SD*=0.9). Finally in the ***Stickiness scale,*** there was a main Group effect (F (1, 48)= 12.97, p<0.001, η^2_G = 0.165) where controls presented greater scores (*M*=2.8 *SD*=1) than meditators (*M*=2. *SD*=0.7). In all graphs, bars denote standard error. Significances *$p < 0.05$, **$p < 0.01$, ***$p < 0.001$.

2.1.5) Alpha-Amylase results:

As Alpha-amylase is secreted in the presence of food as part of the CPRs, we first hypothesized that an increase in Alpha-amylase activity would have taken place in response to the food cue exposure. Secondly, we also hypothesized that this effect would be more pronounced in the Immersed condition compared to the Mindful attention condition.

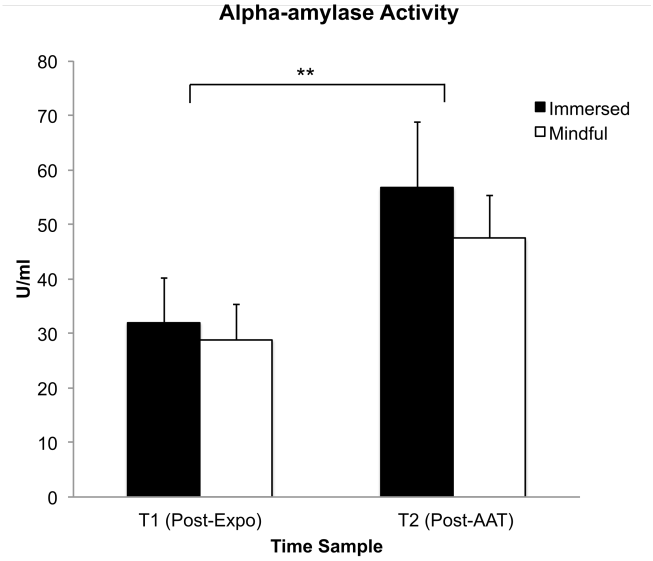

Supplementary figure 6: Alpha-amylase concentration in T1 and T2, after regressing T0 baseline concentration (see Methods), under the two provided instructions. Bars denote standard error. Significances $*p < 0.05$, $**p < 0.01$, $***p < 0.001$

Alpha-amylase activity showed an increase at T1 (*M* =30.5; *SD*=51.5) (t-test, $t(49) = 6.1$, $p<0.001$) and at T2 (*M* =52.1; *SD*=71.5) (t-test, $t(49) = 7.9$, $p<0.001$) compared to T0 (baseline, before exposure), demonstrating the sensitivity of Alpha-amylase activity to our experimental manipulation. To explore Group and Instruction type effects over salivary Alpha-amylase activity we conducted a Group x Instruction type x Time sample (T1/T2) repeated-measure ANOVA. We found a main effect of Time sample ($F(1,48) = 4.9$, $p<0.05$, $\eta^2_G = .006$) driven by higher Alpha-amylase activity at T2 (U/mL) at T2 (*M* =52.1; *SD*=46.7) compared to T1 (*M* = 30.4; *SD*=35.0, t-test, $t(49) = 3.8$, $p<0.01$). Contrary to our hypothesis, neither Group nor Instruction factors modulated this measure. To summarize, while Alpha-amylase activity was sensitive to the Passive vs. Active manipulation of our task, it was not sensitive to our Instruction manipulation (Mindful attention vs. Immersed).

The current finding clearly ruled out Hypothesis 2: the degree of subjective realism does not impact alpha-amylase activity. However, the findings remain inconclusive regarding Hypothesis 1. More specifically, in line with Hypothesis 1, the increase from T0 to T1, could be viewed as reflecting food intake preparation. This interpretation is coherent with the effects of real food intake on Alpha-Amylase[6]. Alpha-Amylase was shown to increase in response to a standardized lunch in stimulated saliva of n = 18 healthy human subjects[6] and to be positively associated with self-ratings of satiety and fullness, and inversely associated with hunger and desire to eat[7]. Importantly, Alpha-amylase was also found to increase within 15 min after eating a standardized meal or ‘‘sham-eating’’ (chewing and expectorating) (n = 12 healthy men and women), even if the increase was significantly lower in the sham- eating condition[8].

Against this interpretation the enhanced alpha-amylase activity from T0 to T1 could merely reflect enhanced sympathetic system activation (see for instance [9]-[10]). This alternative hypothesis is plausible at the light of the enhanced alpha-amylase activity from T2 to T1, which could be also explained along this line. Such increase would be predicted if one assumes that passively viewing food images during the passive exposure phase engages

less the sympathetic system than actively doing a behavioral akin to the AAT. Future studies, will be required to explore these two alternative interpretations, for instance by comparing changes in alpha-amylase activity while immersing in non-food images compared to food images.

2.2) State and Trait Integrative Analyses:

2.2.1) FAB in Relation to Dereification as a Trait Questionnaire:

**Dep. Variable**	**FAB**	**FAB**
Intercept	64.9(34.1)	-11.2(13.3)
Instruction: Mindfulness	-89.1(48.3)	22.4(18.9)
Cognitive Fusion		1.3(0.63)*
Dereification_Trait	-0.96(0.7)	
Instruction X Cognitive Fusion	1.32(0.92)	
Instruction X Dereification_Trait		-2.1(0.89)*
**Model P-value**	**0.005**	**0.001**
**Adjusted R squared**	**0.095**	**0.12**

Supplementary Table 1: Table presenting two different regression models exploring the relation of Dereification as a trait, Cognitive Fusion, and the instruction. Each column presents one model predicting FAB. We present all coefficients estimated followed by its standard error. * $p<0.05$, **$p<0.01$,***$p<0.001$

2.3) Trait Measures:

2.3.1) Trait Integrative Analysis:

As food craving as a trait correlated with AAT performances, we tested whether food craving scores were related to other trait characteristics of Instruction types (Dereification, Cognitive Fusion and BMI). As expected, food craving as a trait was positively correlated with BMI (Pearson r = 0. 2; $p<0.05$) (Fig. Supp 7a) and was also

positively correlated with Cognitive Fusion Questionnaire scores (Pearson r = 0.5, *p*<0.001). Participants who reported greater Cognitive Fusion as a trait, also had higher scores of food cravings as a trait (Fig. Supp 7b). Moreover, food craving was negatively correlated with Dereification Questionnaire scores (Pearson r = -0.6; *p*<0.001) (Fig. Supp. 7c). To summarize, the more a participant reported food craving as a trait, the higher their BMI was, accompanied by a low tendency to dereify perception and a high propensity for cognitive fusion.

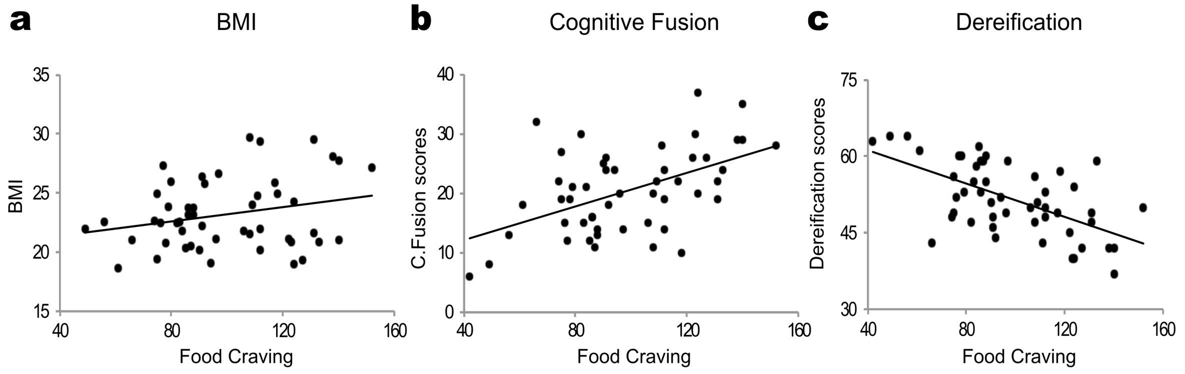

Supplementary figure 7: Trait integrative analyses: a) Food craving as a trait was positively correlated with (BMI), (Pearson r = 0. 2; *p*<0.05), **b)** positively correlated with cognitive fusion questionnaire scores (Pearson r = 0.5, *p*<0.001), **c)** and negatively correlated with the Dereification-as-a-Trait Questionnaire scores (Pearson r = -0.6; *p*<0.001).

2.3.2) Trait Integrative Analysis by Group:

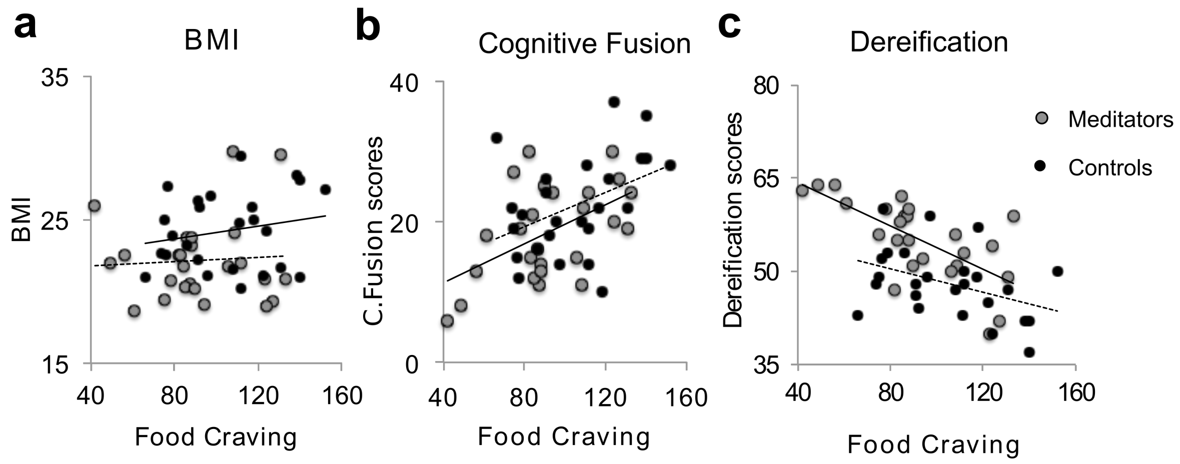

Supplementary figure 8: Trait integrative analysis: Pearson correlations of trait food craving scores with body mass index (BMI), trait Cognitive fusion and Dereification scores, displayed for each group. **a)** Food craving as a trait was not correlated with (BMI), neither for controls (r = 0. 2, *p* =0.2) nor for meditators (r = 0. 12, *p* =0.2). **b)** Trait Food craving scores were positively correlated with Cognitive Fusion scores for both controls (r = 0.4, *p*<0.05) and meditators (r = 0.5, *p* <0.01). **c)** Trait Food craving scores were negatively correlated with the Dereification-as-a-Trait Questionnaire scores for both controls (r = -0.4, *p*<0.05) and meditators (r = -0.7, *p* <0.001).

References

1. Baer, R. A. *et al.* Using Self-Report Assessment Methods to Explore Facets of Mindfulness. *Assessment* (2006). doi:10.1177/1073191105283504

2. Schmidt, Carlos & Eugenia, V. Five Facet Mindfulness Questionnaire: spanish validation. 14–16 (2013).

3. Fabrigar, L. R., Wegener, D. T., MacCallum, R. C. & Strahan, E. J. Evaluating the use of exploratory factor analysis in psychological research. *Psychol. Methods* **4,** 272–299 (1999). doi:10.1037/1082-989X.4.3.272.

4. Costello, A. B. & Osbourne, J. W. Best practices in exploratory factor analysis: Four recommendations for getting the most from your analysis. *Pract. Assessment, Res. Eval.* **10,** 1–9 (2005). doi:10.1.1.110.9154.

5. Papies, E. K., Barsalou, L. W. & Custers, R. Mindful attention prevents mindless impulses. *Soc. Psychol. Personal. Sci.* **3,** 291–299 (2012). doi:10.1177/1948550611419031.

6. Rohleder, N. & Nater, U. M. Determinants of salivary ??-amylase in humans and methodological considerations. *Psychoneuroendocrinology* **34,** 469–485 (2009). doi:10.1016/j.psyneuen.2008.12.004.

7. Harthoorn, L. F. & Dransfield, E. Periprandial changes of the sympathetic-parasympathetic balance related to perceived satiety in humans. *Eur. J. Appl. Physiol.* **102,** 601–608 (2008). doi:10.1007/s00421-007-0622-5.

8. Messenger, B., Clifford, M. N. & Morgan, L. M. Glucose-dependent insulinotropic polypeptide and insulin-like immunoreactivity in saliva following sham-fed and swallowed meals. *J. Endocrinol.* **177,** 407–412 (2003). doi:10.1677/joe.0.1770407.

9. Nater, U. M. & Rohleder, N. Salivary alpha-amylase as a non-invasive biomarker for the sympathetic nervous system: Current state of research. *Psychoneuroendocrinology* **34,** 486–496 (2009). doi:10.1016/j.psyneuen.2009.01.014.

10. Bosch, J. A., Veerman, E. C. I., de Geus, E. J. & Proctor, G. B. A-Amylase As A Reliable And Convenient Measure Of Sympathetic Activity: Don't start salivating just yet! *Psychoneuroendocrinology* **36,** 449–453 (2011). doi:10.1016/j.psyneuen.2010.12.019.
